# Toward elucidation of genetic and functional genetic mechanisms in corn host resistance to *Aspergillus flavus* infection and aflatoxin contamination

**DOI:** 10.3389/fmicb.2014.00364

**Published:** 2014-07-21

**Authors:** Xueyan Shan, W. Paul Williams

**Affiliations:** ^1^Department of Biochemistry, Molecular Biology, Entomology, and Plant Pathology, Mississippi State UniversityMississippi, MS, USA; ^2^Agricultural Research Service, United States Department of Agriculture, Corn Host Plant Resistance Research UnitMississippi, MS, USA

**Keywords:** *Aspergillus flavus*, aflatoxin, corn, host resistance, transcriptomics

## Abstract

Aflatoxins are carcinogenic mycotoxins produced by some species in the *Aspergillus* genus, such as *A. flavus* and *A. parasiticus*. Contamination of aflatoxins in corn profusely happens at pre-harvest stage when heat and drought field conditions favor *A. flavus* colonization. Commercial corn hybrids are generally susceptible to *A. flavus* infection. An ideal strategy for preventing aflatoxin contamination is through the enhancement of corn host resistance to *Aspergillus* infection and aflatoxin production. Constant efforts have been made by corn breeders to develop resistant corn genotypes. Significantly low levels of aflatoxin accumulation have been determined in certain resistant corn inbred lines. A number of reports of quantitative trait loci have provided compelling evidence supporting the quantitative trait genetic basis of corn host resistance to aflatoxin accumulation. Important findings have also been obtained from the investigation on candidate resistance genes through transcriptomics approach. Elucidation of molecular mechanisms will provide in-depth understanding of the host–pathogen interactions and hence facilitate the breeding of corn with resistance to *A. flavus* infection and aflatoxin accumulation.

## INTRODUCTION

Aflatoxins are produced by *Aspergillus flavus*, an ear rot fungus that infects corn. Aflatoxins are carcinogenic to humans and animals. Consequently, they are of great concern as food contaminants and are closely monitored in oily-seeded crops and dairy products (Payne, 1992; Gourama and Bullerman, 1995). Aflatoxins are designated as B1, B2, G1, G2, M1, and M2 types based on their molecular structures. Aflatoxin B1 is the most toxic form which is found in fungal-infected corn kernels (Bhatnagar et al., 2006). The production of aflatoxin B1 starts soon after the colonization of *A. flavus* in the developing corn kernels (Thompson et al., 1983). Accumulation of aflatoxin B1 continues during the maturation of corn. Commercial corn hybrids are generally susceptible to *A. flavus* infection. The Food and Drug Administration (FDA) has imposed a strict limit of 20 ppb on aflatoxin concentration present in corn (Aflatoxin Guidelines, ). Aflatoxins remain stable under food processing conditions. Once corn is contaminated with aflatoxins, few detoxification options are available. Therefore, the prevention of aflatoxin accumulation should start in pre-harvest corn stage.

*Aspergillus flavus* infection and aflatoxin accumulation in pre-harvest corn largely depend on the local weather and field conditions. High temperature and drought conditions are in favor of aflatoxin production, thus posing the high risk of an aflatoxin outbreak. The colonization of *A. flavus* also frequently occurs at sites damaged by insects. In many locations of the southeastern United States, including Mississippi, *A. flavus* infection in corn is a chronic problem. In 1998, a severe aflatoxin outbreak in corn fields occurred in Mississippi and throughout the Southeast, resulting in considerable economic loss to corn farmers (Robens and Cardwell, 2005). In Mississippi, efforts to reduce aflatoxin contamination in corn at the pre-harvest stage have focused on multiple strategies including bio-control using atoxigenic *A*. *flavus* strains, optimal agronomic practices (irrigation, fungicides, planting dates), and breeding for host resistance (Larson, 1997; Cleveland et al., 2004). While notable improvement can be achieved through optimizing cultural practices for control of pre-harvest aflatoxin contamination, combination strategy by including breeding for corn host resistance would be the most promising and effective avenue.

## RESISTANT CORN GERMPLASM DEVELOPED BY USDA–ARS AT MISSISSIPPI STATE

As early as in the 1970s, USDA–ARS scientists at Mississippi State University initiated breeding programs for screening and developing corn germplasm with resistance to *A. flavus* infection and aflatoxin accumulation. Reliable techniques (such as side-needle inoculation) were first developed to assure that all germplasm lines were treated with the same amount of fungal inoculums (Zummo and Scott, 1989; Windham et al., 2005, 2009; Buckley et al., 2006). Numerous germplasm and breeding lines were screened with such consistent methods. The results of these evaluations established the foundation of today’s collection of Mississippi corn inbred lines that possess naturally occurring resistance to aflatoxin accumulation (Windham and Williams, 1998, 1999, 2002; Windham et al., 1999, 2010; Williams et al., 2002, 2005a,b, 2008; Williams, 2006). Resistant corn inbred lines were developed by consecutive selfing and selection against aflatoxin accumulation. In 1988, the first inbred line Mp313E with resistance to *A. flavus* infection was released (Scott and Zummo, 1990). Mp313E was developed from germplasm Tuxpan. Since the release of Mp313E, additional resistant corn germplasm lines (Mp420, Mp715, Mp717, Mp718, and Mp719) have also been released; all exhibit significantly low levels of aflatoxin accumulation under artificial inoculation conditions (Scott and Zummo, 1992; Williams and Windham, 2001, 2006, 2012). Mp715, Mp718, and Mp719 also have lineages tracking back to germplasm Tuxpan. Tuxpan is a US derivative of Tuxpeño, one of the most productive and successful Mexican races of corn.

In the continual field studies conducted under artificial inoculation conditions, aflatoxin levels were evaluated for selected resistant corn inbred lines, single-cross hybrids from the selected resistant corn inbred lines, and a set of commercial corn hybrids. In 1999, aflatoxin level in the resistant corn inbred line Mp715 was 24 ppb which was contrasted to the level of 1622 ppb in SC212m, a susceptible corn inbred line. In 1996, aflatoxin level in the single-cross hybrid Mp715 × Mp313E was 18 ppb compared to 1532 ppb in the single cross of GA209 × SC212m. In 2001, aflatoxin level for Mp715 was 14 ppb compared to 9289 ppb for SC212M. In 2008, aflatoxin level was as low as 17 ppb for the single-cross hybrid of Mp313E × Mp717, whereas it was as high as 10800 ppb for a commercial hybrid. Similar results were observed in 2009, where aflatoxin levels ranged from 5 ppb for the single-cross hybrid of Mp313E × NC388 to 1992 ppb for a commercial hybrid. From these studies, the lowest aflatoxin levels were found in the single-cross hybrids of the resistant inbred lines developed by the USDA–ARS Corn Host Plant Resistance Research Unit (CHPRRU) at Mississippi State. Four of the single-cross hybrids had aflatoxin levels lower than the FDA action level of 20 ppb in the 2008 and 2009 field studies. Commercial corn hybrids generally contained consistently high levels of aflatoxins in the studies conducted in 2008 and in 2009 at Mississippi State (Daves et al., 2010).

## THE IDENTIFICATION OF MAJOR RESISTANCE QUANTITATIVE TRAIT LOCI (QTLs)

**FIGURE 1:**
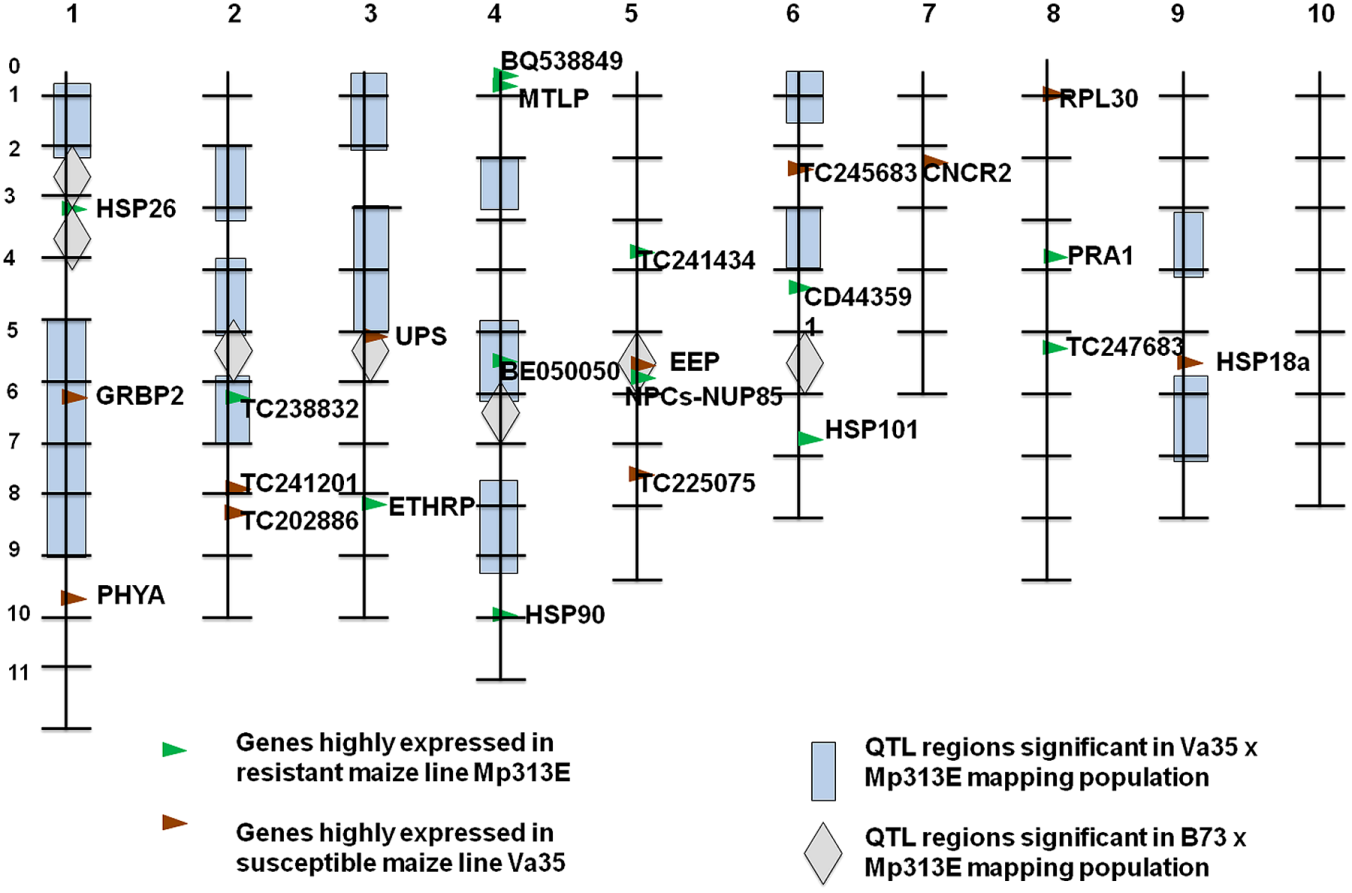
**Chromosome bin map showing the positions of the identified QTL regions from the two Mp313E-derived QTL mapping populations.** The significant genes marked in the map were from a separate genome-wide microarray study. Eight genes were located to seven most significant QTL regions based on the microarray study. A significant candidate gene, NPCs–NUP85, is located to the chromosome 5 QTL region (Chr 5, bin 5.05) which is from Mp313E (Kelley et al., 2012).

The resistant corn inbred lines exhibited significant general combining ability for reduced aflatoxin accumulation in a diallel cross, indicating good breeding values for the resistance trait (Williams et al., 2007). To incorporate the resistance into commercially acceptable lines for corn farmers, CHPRRU has made important progress in combining traditional and molecular breeding methods to develop resistant corn lines. Among the resistant corn germplasm lines developed by CHPRRU at Mississippi State, Mp313E and Mp715 are two of the most important parental lines used for breeding of *A. flavus* resistance and aflatoxin reduction. They have been used as resistance donors for many of the new experimental lines in the CHPRRU breeding programs. Two quantitative trait loci (QTL) mapping populations with Mp313E as a parental line were developed and investigated. The population from Mp313E × Va35 included 216 F_2:3_ families and was evaluated for aflatoxin accumulation over 3 years (Davis et al., 2000; Willcox et al., 2013). A total of 15 QTL regions were identified (**Figure 1**). Four QTLs on chromosomes 1, 4, and 9 were above a significance level of 23.58 in likelihood ratio. The chromosome 4 (bin 4.04–4.08) QTL was associated with Mp313E. The Mp313E × B73 population contains 210 F_2:3_ families and was also evaluated for 3 years (Brooks et al., 2005). A total of seven QTL regions were identified (**Figure 1**). The two QTLs on chromosomes 2 and 4 were most significant and were associated with Mp313E. From these studies, up to 48% of the genotypic effects of the resistance can be explained by the QTLs identified on chromosomes 2, 3, and 4 from Mp313E. Significant progress has been made to increase corn host plant resistance by DNA marker-assisted breeding. However, a major obstacle has been that the genomic regions containing the QTLs are large and indecipherable without the knowledge of the underlying genetic and molecular information. Closely linked molecular markers to the QTLs, ideally from resistance genes, are needed to expedite the breeding process and to reduce the breeding cycles.

## COMPARATIVE GENOME-WIDE GENE EXPRESSION STUDY ON SELECTED CORN INBRED LINES

Corn host resistance to *A. flavus* infection and aflatoxin accumulation is a quantitative trait potentially controlled by many genes. Characterization of the corresponding genes and their effects is essential for the breeding of resistant corn lines. To characterize genes involved in the corn host plant resistance, a microarray study was conducted to investigate gene expression patterns in two resistant corn inbred lines (Mp313E and Mp04:86) and two susceptible corn inbred lines (Va35, B73) under artificial inoculation in field conditions (Kelley et al., 2012).

Developing kernels of corn were collected from field for RNA preparation. The field experimental plan was a randomized, complete block design with split plots and three replications. The average aflatoxin levels in mature corn kernels from the inoculated primary ears of each genotype were measured as 195 ppb for Mp04:86, 140 ppb for Mp313E, 1243 ppb for Va35, and 3791 ppb for B73. Mp04:86 was a resistant recombinant inbred line derived from the cross of Mp715 (resistant) × Va35 (susceptible), and it was selected by the phenotypic trait of low-level aflatoxin accumulation. The microarrays (from the NSF Maize Oligonucleotide Array Project) used in this experiment contained 57,452 maize gene probes (Pontius et al., 2003).

**FIGURE 2:**
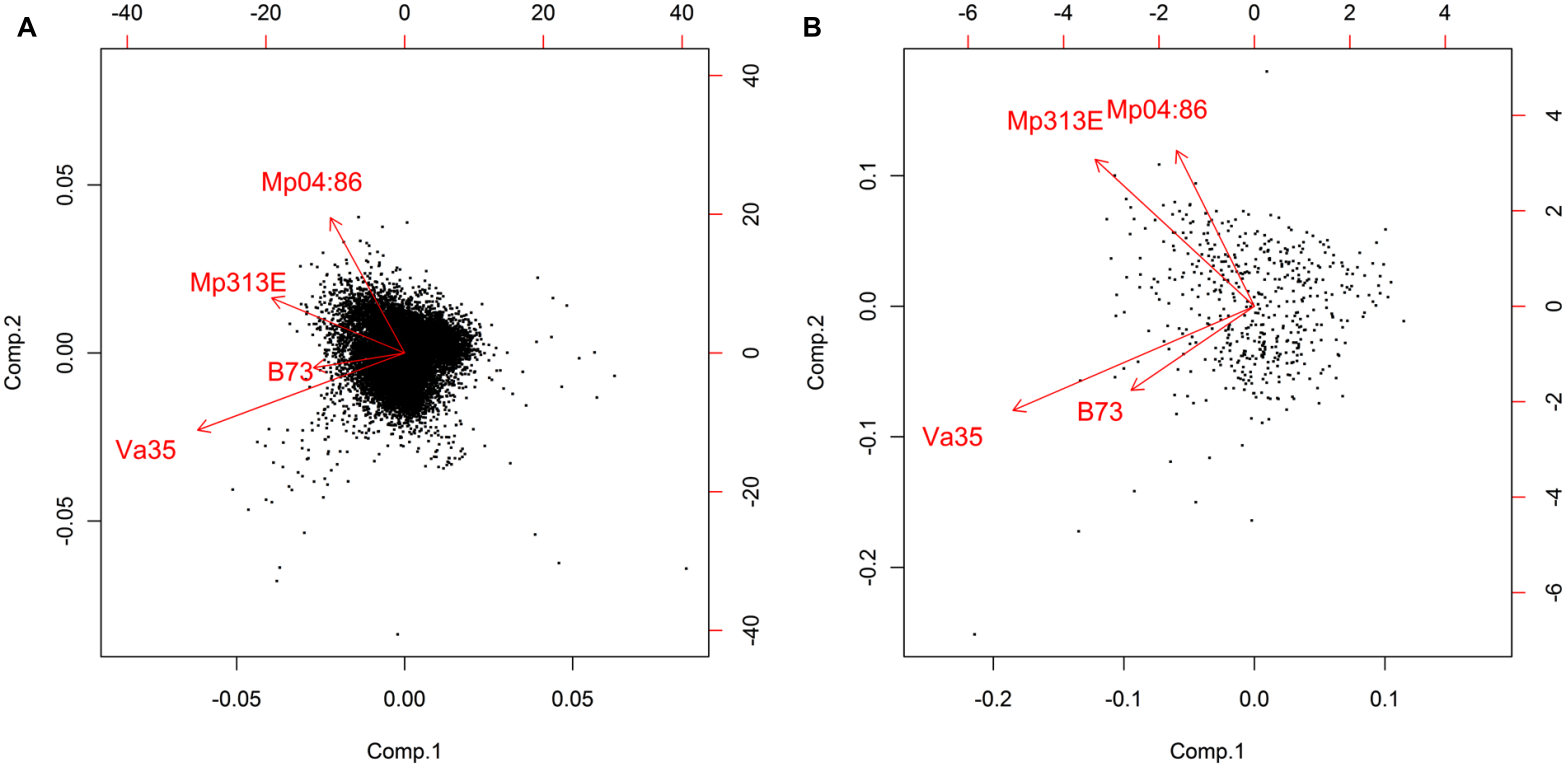
**Biplots of PCA on the log2 ratios (*I/U*) of expressed genes in corn inbred lines.** The eigenvectors evidently separated into resistant and susceptible groups, respectively. Genes coordinately expressed in resistant lines located near the eigenvectors for Mp313E and Mp04:86. Genes coordinately expressed in susceptible lines located near the eigenvectors for Va35 and B73. **(A)** PCA of 13,107 expressed genes and **(B)** PCA of a subset of 500 expressed genes. The arrows represent eigenvectors. The direction and length represent the larger variance of the fold changes (Kelley et al., 2012).

The log2 fold changes (inoculation/un-inoculation) for each gene probe were used as gene expression values, resulting in a high-dimensional (57,452 × 4) data matrix. Principal component analysis (PCA) was used to analyze the resulting gene expression values. PCA is a commonly used technique in finding patterns and relationships between variables in data analysis and the results can be displayed in biplots. The direction and length of the arrows in the biplots represented the larger variances in the gene expression values of each corn inbred line (**Figures 2A,B**). By using PCA analysis, the resistant corn inbred lines (Mp313E and Mp04:86) were separated from the susceptible corn inbred lines (Va35 and B73). It had important implications. The patterns of gene expression appeared to be similar in Mp313E and Mp04:86 in contrast to those of Va35 and B73. The fact that Mp04:86 was clustered with Mp313E (resistant, not a parent) but not with the susceptible parent Va35 strongly indicated that Mp313E and Mp04:86 might have shared similar resistance mechanisms. It is worth mentioning that the PCA analysis was directly applied to all gene expression values without any pre-selection by statistical significance levels. These findings demonstrated that all levels of the gene effects collectively contributed to the grouping of resistant genotypes versus susceptible genotypes.

The microarray data were also analyzed statistically to determine the significance levels and the significant genes were selected for further validation by quantitative real-time PCR (qRT-PCR) experiments. Fifty genes that were statistically significant from microarray study were selected for a qRT-PCR analysis in a separate time course experiment using developing kernel samples of Mp313E and Va35. Thirty-one of the 50 genes were found to be significantly differentially expressed (*P* < 0.05) by analysis of qRT-PCR data. Among these genes, eight were mapped to seven previously identified QTL regions (**Figure 1**; Kelley et al., 2012).

Three of the mapped significant genes (**Figure 1**) that were highly expressed in Va35 were known plant stress responsive genes. AI664980 encodes a glycine-rich RNA-binding protein (GRBP2; Naqvi et al., 1998; Govrin and Levine, 2000; Singh et al., 2011). TC234808 encodes a protein that governs the large and small ribosomal subunits assembly. Both AI664980 and TC234808 genes have RNA-binding domains and are likely to be involved in the post-transcriptional regulation in plant defense systems. BG266083 encodes stress-induced small heat shock proteins (sHSPs). The development of ear rot caused by *A. flavus* infection in the susceptible corn inbred line Va35 showed hypersensitivity. AI664980 (GRBP2) protein was reported to be associated with plant–pathogen hypersensitivity interactions (Naqvi et al., 1998).

The resistant corn inbred line Mp313E appeared to have highly expressed genes encoding RNA transport regulators, molecular chaperones, and detoxification proteins. TC231674 is homologous to the human nucleoporin NUP85 which is a component of the nuclear pore complexes (NPCs). Studies have strongly suggested that components of NPCs regulate the transport of R proteins (Cheng et al., 2009; Garcia and Parker, 2009; Faris et al., 2010). TC231674 gene was found highly expressed in the resistant line Mp313E. BM498943 encodes ethylene responsive protein (ETHRP) which belongs to the universal stress responsive protein family. BM379345 encodes a metallothionein-like protein (MTLP) involved in the detoxification of heavy metal ions. It was found that increased aflatoxin production was associated with high levels of certain trace metal elements (Lillehoj et al., 1974).

## EXPLORATION OF COMPUTATIONAL AND STATISTICAL METHODS ON ANALYSIS OF RNA PATHWAY GENE EXPRESSION DATA

Evidence from the microarray study showed that TC231674, which encodes a possible RNA transport regulator, was highly expressed in the resistant corn inbred line Mp313E. A number of previous studies have demonstrated that RNA transport pathway genes play direct roles in plant defense (Piffanelli et al., 1999; Chisholm et al., 2006; Walley et al., 2007). RNA transport pathways are made up from a number of different protein complexes. For example, the cap binding complex (CBC), spliceosome, transcription-export complex (TREX), exon-junction complex (EJC), and translation initiation factors (eIFs) are involved in the transport of mRNA (Nakielny et al., 1997). Importins, exportins, Ran-GTP-related protein complex, and the survival motor neuron complex (SMN) are for the transport of rRNA, tRNA, and snRNA molecules (Kindler et al., 2005; Jambhekar and Derist, 2007; Vazquez-Pianzola and Suter, 2012). All RNAs are transported across the nuclear membrane through NPCs (Köhler and Hurt, 2007; Meier and Brkljacic, 2009; Deslandes and Rivas, 2011; Rivas, 2012). Several reports have shown that nucleoporins are directly involved in the regulation of R protein activities.

The expression patterns of corn RNA transport pathway genes and their relations were studied by a qRT-PCR experiment in the selected resistant and susceptible corn inbred lines (Asters et al., 2014). The advance of qRT-PCR technique makes it possible to precisely describe and compare the level of gene expression changes (Heid et al., 1996; Kubista et al., 2006; Rieu and Powers, 2009). In contrast to the comprehensive genome-wide microarray or RNA sequencing techniques, qRT-PCR provides the flexibility to measure gene expression levels on more samples across a wide range of experimental conditions as “phenotypic traits.” This is an important and novel strategy since it presents a way to overcome the commonly seen “phenotyping bottleneck” due to the limitation of available measureable traits compared with the huge amount of genomic single nucleotide polymorphisms (SNPs) data. We aimed at the exploration on using gene expression values as the “phenotypic data” in addition to the levels of aflatoxin accumulation to conduct future genetic marker analysis.

Experiments were conducted to establish a computational pipeline for analysis of qRT-PCR gene expression data (Asters et al., 2014). This research aimed at the qRT-PCR gene expression analysis of more corn inbred lines, focused pathways, with an experimental design in parallel to a typical breeding project, so that statistical analysis over potential DNA markers, candidate gene expression levels, and aflatoxin levels could directly apply. Resistant (Mp718, Mp719, and Mp04:104) and susceptible (Va35, Mp04:85, and Mp04:89) corn inbred lines were used in this experiment. Experimental conditions consisted of two treatments (inoculated and un-inoculated with *A. flavus*), six corn inbred lines with three replications for each, and two sample collection time points at 2 and 7 days after inoculation (DAI). Since these inbred lines were offspring from a single cross of Mp715 and Va35, only up to two possible alleles for each gene were involved in all samples. The gene expression variations observed in these samples were likely related to gene regulating patterns associated with the resistance or susceptibility.

**FIGURE 3:**
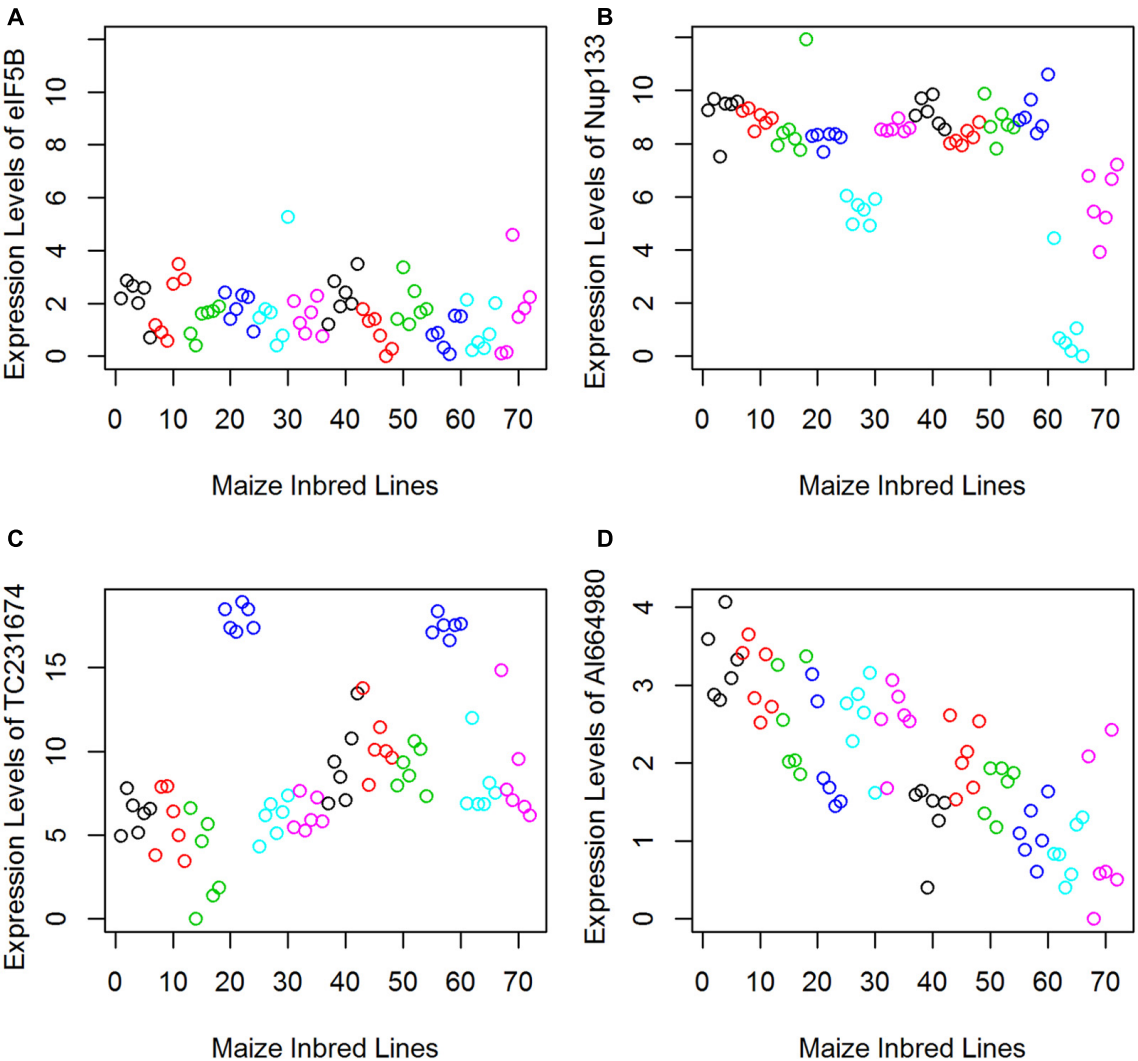
**Examples **(A–D)** showing that qRT-PCR gene expression values can be used as “phenotypic data” for statistical analysis.** Each scatterplot represents gene expression values measured for one gene across all samples. The horizontal axis represents the 72 samples with different colors coded for the six corn inbred lines. Samples 1–36 were collected at 2 DAI and samples 37–72 were at 7 DAI. The vertical axis represents the relative gene expression values (Asters et al., 2014).

Forty RNA transport pathway genes and 16 candidate genes identified from the previous microarray experiment were investigated. Trends of gene expression patterns were observed among the test corn inbred lines (**Figure 3**). AI664980 showed significant variations among samples with down-regulation patterns in resistant lines. TC231674 was found highly expressed in the resistant corn line Mp718. Of the 56 genes analyzed, 17 were significantly differentially expressed between the resistant and the susceptible groups. Most of the significant genes were from the NPC and SMN protein complexes. Resistance candidate genes BE050050, TC231674, and BM498943 were found positively correlated. Whereas the susceptibility-related gene AI664980 was found negatively correlated with BE050050 (-0.97; Asters et al., 2014). The inclusion of previously identified candidate genes in this research provided another way of validation. The observation was consistent with the previous findings on TC231674 where it was found highly expressed in a different resistant corn inbred line Mp313E.

The genes from the RNA transport pathways were considered as being selected from a static gene network. However, in contrast to the methods excessively used in the functional clustering by matching with Gene Ontology (GO) terms, the analysis in this study aimed to explore a method on determination of gene relationships from the empirical gene expression data. Susceptibility-related gene AI664980 was found clustered with six test genes (Nup88, eIF2, CD443591, CA399536, SPN1, and MAGOH) by a network analysis method, suggesting a similar expression pattern of these genes in response to *A. flavus* infection. Resistance candidate genes, such as TC231674 and BE050050, appeared as isolates in the network. Further experiments with more genes included are needed to reveal positions of these genes in the empirical network regarding corn defense against *A. flavus* infection and toxin production (Asters et al., 2014).

## CONCLUSION

Many studies have shown that significantly low levels of aflatoxin accumulation have been achieved in a number of resistant corn inbred lines. However, an efficient transfer of such resistance to commercially available corn lines has been proven difficult due to the complex nature of this quantitative trait. Many genes were proposed to be involved in the corn host plant resistance to the aflatoxin reduction. The exploration for effective methods to identify and prioritize resistance genes will expedite the discovery of DNA markers and facilitate the breeding for corn host resistance.

For future work, we propose the use of multi-environmental gene expression data as “phenotypic data” for calculation of the DNA marker genetic effects in performing genomic selection for the breeding of corn host resistance. Parallel quantitative proteomics and metabolomics approaches will also be explored to identify proteins and metabolites related to *A. flavus* responsive pathways. Using transcriptomics, proteomics, and metabolomics data as “phenotypic data” will facilitate the identification of connections among genome regions, resistance-related genes and proteins, aflatoxin contents, and other metabolites for statistical analysis and modeling. The application of the resulting statistical models will be very helpful on estimating marker genetic effects and predicting the breeding values for aflatoxin resistance in corn.

## Conflict of Interest Statement

The authors declare that the research was conducted in the absence of any commercial or financial relationships that could be construed as a potential conflict of interest.
